# Effects of swimming activity and feed restriction on antioxidant and digestive enzymes in juvenile rainbow trout: Implications for nutritional and exercise strategies in aquaculture

**DOI:** 10.1002/vms3.1466

**Published:** 2024-05-02

**Authors:** Gökhan Tunçelli, Onur Ertik, Bertan Boran Bayrak, Devrim Memiş, Refiye Yanardag

**Affiliations:** ^1^ Department of Aquaculture and Fish Diseases Faculty of Aquatic Sciences Istanbul University Istanbul Turkey; ^2^ Department of Chemistry Faculty of Engineering Istanbul University‐Cerrahpaşa Istanbul Turkey

**Keywords:** animal welfare, enzyme activity, exercised fish, restricted feeding, recirculating aquaculture systems

## Abstract

**Background:**

In this study, we investigated the effects of swimming activity and feed restriction on digestion and antioxidant enzyme activities in juvenile rainbow trout (average body weight of 26.54 ± 0.36 g).

**Methods:**

The stomach, liver and kidney tissues were obtained from four distinct groups: the static water group (fish were kept in static water and fed to satiation), the feeding restricted group (fish were kept in static water with a 25% feed restriction), the swimming exercised group (fish were forced to swimming at a flow rate of 1 Body Length per second (BL/s)) and the swimming exercised‐feed restricted group (subjected to swimming exercise at a 1 BL/s flow rate along with a 25% feed restriction). We determined the levels of glutathione, lipid peroxidation and the activities of catalase, superoxide dismutase, glutathione peroxidase, glutathione reductase, glucose‐6‐phosphate dehydrogenase and lactate dehydrogenase, as well as the presence of reactive oxygen species in the tissues obtained from the fish. Additionally, the activities of pepsin, protease, lipase and arginase in these tissues were measured.

**Results:**

Swimming activity and feed restriction showed different effects on the enzyme activities of the fish in the experimental groups.

**Conclusion:**

It can be concluded that proper nutrition and exercise positively influence the antioxidant system and enzyme activities in fish, reducing the formation of free radicals. This situation is likely to contribute to the fish's development.

## Graphical abstract

This research investigates the impact of swimming activity and feed restriction on the antioxidant and digestive enzyme activities in juvenile rainbow trout, revealing differential effects across various enzyme activities. Key findings demonstrate that tailored exercise and nutritional regimens can significantly enhance the antioxidant defense system, potentially reducing free radical formation and supporting fish development in aquaculture settings. These insights highlight the importance of balanced exercise and nutrition strategies in aquaculture to promote fish health and growth.

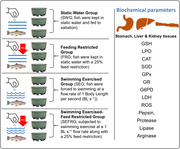

## 1 INTRODUCTION

Regularly applied physical exercises can have both positive and detrimental effects on organisms. One of the negative consequences includes the formation of free radicals and lipid peroxidation (LPO) due to exercise (Gonenc, 1995). The intensity of exercise directly affects the generation of free radicals, with higher intensity exercises resulting in more pronounced free radical formation (Palmer et al., 2003). The literature provides evidence of increased reactive oxygen species (ROS) and free radical formation during intense exercise, leading to oxidative damage in muscles, liver, blood and other tissues (König et al., 2001; Urso & Clarkson, 2003). Regular and prolonged exercises are suggested to strengthen the protective system termed the ‘antioxidant defence system’ and to reduce LPO products (Gul et al., 2001; Ji, 1999). Properly conducted physical exercises can contribute to maintaining a healthy lifestyle (Vincent et al., 2002).

The intensity and duration of swimming in fish affect enzyme activities (Melzner et al., 2009). Similar to other vertebrates, fish possess an antioxidant defence system responsible for counteracting the effects of hydrogen peroxide and ROS, which cause oxidative damage to cellular molecules such as fats, proteins and DNA, leading to cell death (Yonar & Sakin, 2011). This antioxidant defence system comprises enzymes such as superoxide dismutase (SOD), catalase, glutathione peroxidase (GPx), glutathione‐S‐transferase (GST), glucose‐6‐phosphate dehydrogenase (G6PD) and glutathione reductase (GR) (Blahová et al., 2013), as well as non‐enzymatic antioxidants, such as reduced glutathione, vitamin E, vitamin C, melatonin and flavonoids (Droge, 2002). These enzymatic and non‐enzymatic systems function to scavenge free radicals and prevent the formation of ROS that cause oxidative damage (Aydın et al., 2001; Li et al., 2009). An imbalance resulting from a decrease in antioxidant enzyme activities and/or excessive accumulation of ROS, due to the quantities of antioxidants and oxidants, can lead to oxidative stress in an organism (Valavanidis et al., 2006). Determining oxidative stress parameters is performed to assess the status of the antioxidant defence system in fish (Sturve et al., 2005). Environmental conditions, feeding habits and dietary components influence the antioxidant defence system in fish. Generally, antioxidant defence mechanisms are more prominent in the liver tissue than in other tissues such as the kidney and brain (Li et al., 2015). Assessing the activities of antioxidant enzymes is crucial for evaluating fish health (Sagstad et al., 2007).

In this study, the effects of feeding and physical activities on antioxidant enzyme activities in fish tissues (stomach, liver and kidney), as well as the impact of physical activity on lactate dehydrogenase (LDH) activity in all tissues, were determined in a recirculating aquaculture system (RAS). The alterations in enzyme activities have helped in assessing whether the growth and farming performance of fish raised in RAS are positively affected. The main focus of this study was to compare the digestive and antioxidant enzyme activities in rainbow trout subjected to continuous swimming against a current and feeding restriction. Determining these enzyme activities is essential for understanding how swimming and feeding restrictions affect fish health in aquaculture practices. This study investigated the relationship between swimming activity and feeding restriction on the digestive and antioxidant enzyme activities in fish tissues obtained from the RAS experimental groups.

## 2 MATERIALS AND METHODS

### 2.1 Experimental groups

The study examined the effects of both current presence and feed restriction, with the study groups designed as follows:

Static water group (SWG): This group was kept in static water (no current, 0 BL/s) and fed to satiation (∼2% of fish weight).

Feeding restricted group (FRG): This group was kept in static water (no current, 0 BL/s) and with a 25% feed restriction compared to the SWG group.

Swimming exercised group (SEG): This group was engaged in swimming exercise at a 1 BL/s current was fed to satiation (∼2% of fish body weight).

Swimming exercised and feed restricted group (SEFRG): This group engaged in swimming exercise at a 1 BL/s current and with a 25% feed restriction compared to the SEG group.

To create a current in the fish tanks, the methods described by McKenzie et al. (2012) were used. Data pertaining to fish growth and water quality have been previously published in a prior study (Tunçelli & Memiş, 2024).

### 2.2 Experimental design of RAS systems

**FIGURE 1:**
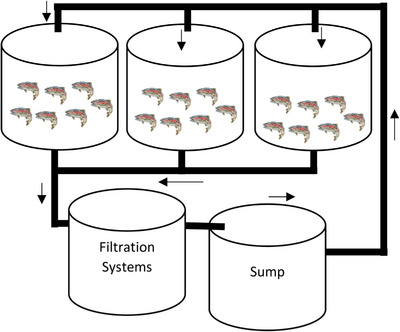
Schematic design of the recirculating aquaculture system (RAS) for four different experimental groups.

Four separate RAS were established for each group involved in the experiments. Each RAS consisted of three fish tanks (see Figure 1), with each tank housing 36 fish weighing 27.8 ± 2.6 g. Random samples of fish were taken from the established groups for the determination of glutathione, LPO and ROS levels, as well as antioxidant enzymes, LDH activity and protein content in their tissues and organs. Additionally, pepsin and protease enzyme activities were measured in their stomachs, whereas lipase enzyme activity was assessed in both stomach and liver tissues, and arginase activity was determined in the liver and kidney tissues.

### 2.3 Enzyme activity analyses

#### 2.3.1 Tissue preparation and biochemical analysis

At the end of the 6 weeks, samples of stomach, liver and kidney tissues were collected. Thereafter, 10% (weight/volume) tissue homogenates were prepared using a cold physiological saline solution. These homogenates were centrifuged at 10.000 × *g* for 10 min at 4°C. Clear supernatants were pooled out for biochemical analysis.

Reduced glutathione (GSH) and LPO levels were estimated according to Beutler (1975) and Ledwozyw et al. (1986) methods, respectively. Catalase (CAT), SOD, GPx, GR, GST, G6PD and LDH activities in all homogenates were assessed by the methods described by Aebi (1984) and Paglia and Valentine (1967) and modified by Wendel (1981), Beutler (1971), Habig and Jakoby (1981), Beutler (1984) and Wroblewski (1957), respectively. ROS levels in the supernatants were quantified following the established procedures delineated by Zhang et al. (2018).

For stomach and liver tissue, pepsin, protease and lipase activity determinations were conducted across a range of pH values reflective of the physiological conditions of the digestive tract. Lipase activity was determined using the Conforti method (2012), with modifications as applicable, whereas pepsin activity was assessed employing the Anson method (1938) with tissue‐specific modifications. Protease activity in gastric tissue was measured using a modified version of the method proposed by Singh et al. (2011).

Arginase activity in the tissue samples was quantified according to the established protocol by Geyer and Dabich (1971). Finally, protein content in the samples was calculated utilizing the Lowry method as detailed by Lowry et al. (1951).

### 2.4 Statistical analysis

The results of the current study were evaluated by using GraphPad Prism Software, version 6.01 (San Diego). Data were stated as mean ± standard deviation by using one‐way analysis of variance followed by Tukey's multiple comparison post hoc test. *p* < 0.05 was considered statistically significant.

## 3 RESULTS

In stomach tissues, GSH and LPO values and GPx and GR activities increased in SWG and SEG groups compared to FRG and SEFRG groups. CAT activity increased in FRG compared to SWG and decreased in SEFRG compared to SEG groups (Table 1). GST activity increased in FRG compared to SWG and decreased in SEFRG compared to SEG groups. SOD activity increased in FRG compared to SWG groups. There were no significant differences in SOD activity in the SEG and SEFRG groups. G6PD activity increased in FRG compared to SWG, and no change in SEFRG compared to SEG group. The ROS value increased in FRG compared to SWG and in SEFRG compared to SEG group. LDH activity decreased in the FRG group and SEFRG group compared to SWG and SEG group (Table 1). Lipase activity decreased in the SWG group compared to the FRG group. There were no significant differences in lipase activity in the SEG and SEFRG groups. Pepsin and protease activities of SWG and SEG groups increased compared to FRG and SEFRG groups.

**TABLE 1:** Biochemical parameters of stomach tissues of all groups.

Biochemical parameters	SWG	FRG	SEG	SEFRG	*p_ANOVA_*
**Glutathione (nmol GSH/mg protein)** ^*^	19.82 ± 1.29^a^	13.38 ± 0.65	17.14 ± 1.56^g^	12.07 ± 1.65	<0.0027
**Lipid peroxidation (nmol MDA/mg protein)** ^*^	6.36 ± 1.99	5.39 + 2.93	7.18 ± 1.97	6.68 ± 0.62	<0.431
**Catalase (U/mg protein)** ^*^	255.80 ± 45.50	286.40 ± 55	288.80 ± 16.8^c^	139.10 ± 17.3^b^	<0.0004
**Superoxide dismutase (U/mg protein)** ^*^	1.36 ± 0.30	1.46 ± 0.32	1.15 ± 0.24	1.14 ± 0.12	<0.2938
**Glutathione peroxidase (U/mg protein)** ^*^	16.28 ± 0.80^e^	12.80 ± 1.97	12.03 ± 1.35^g^, ^h^	9.09 ± 1.86^e^	<0.0001
**Glutathione reductase (mU/g protein)** ^*^	12.69 ± 0.90^d^	5.78 ± 0.60	8.64 ± 2.35^i^	7.81 ± 0.32^b^	<0.0001
**Glutathione‐S‐transferase (U/g protein)** ^*^	0.55 ± 0.10^d^	3.05 ± 0.33	1.13 ± 0.38^j^	1.00 ± 0.43^d^	<0.0001
**Glucose‐6‐phosphate dehydrogenase (U/g protein)** ^*^	1.31 ± 0.34	1.45 ± 0.02	0.80 ± 0.32^j^	0.81 ± 0.50^e^	<0.0047
**Reactive oxygen species (RFU/mg protein)** ^*^	13.16 ± 0.55^e^	15.12 ± 1.52	10.64 + 1.24^g^, ^h^	12.63 ± 0.98^a^	<0.0001
**Lactate dehydrogenase (U/mg protein)** ^*^	1.27 ± 0.14	1.21 ± 0.06	1.39 ± 0.32^c^	0.83 ± 0.14^e^	<0.0009
**Lipase (U/mg protein)** ^*^	0.05 ± 0.02^b^	0.14 ± 0.04	0.15 ± 0.04^[Link]^, ^g^	0.15 ± 0.02	<0.0001
**Pepsin (U/mg protein)** ^*^	122.30 ± 1.55^e^	91.18 ± 3.33	133.10 ± 12.24^f^	101.40 ± 3.59	<0.0007
**Protease (U/mg protein)** ^*^	0.45 ± 0.11^e^	0.28 ± 0.03	0.79 ± 0.09^[Link]^, ^f^	0.56 ± 0.08^b^	<0.0001

Abbreviations: ANOVA, analysis of variance; FRG, Feeding restricted group; SEFRG, swimming exercised‐feed restricted group; SEG, swimming exercised group; SWG, static water group.

* Mean ± SD.

^a^ *p* < 0.01 vs. FRG.

^b^ *p* < 0.001 vs. FRG.

^c^ *p* < 0.001 vs. SEFRG.

^d^ *p* < 0.0001 vs. FRG.

^e^ *p* < 0.05 vs. FRG.

^f^ *p* < 0.01 vs. SEFRG.

^g^ *p* < 0.05 vs. SEFRG.

^h^ *p* < 0.01 vs. SWG.

^i^ *p* < 0.05 vs. SWG.

^j^ *p* < 0.05 vs. SWG.

It was determined that the GSH value and GPx and GST activities were decreased in the FRG and SEFRG groups compared to the SWG and SEG groups, and the LPO value was decreased in the SEG group compared to the SEFRG group. There were no significant differences in LPO levels in the SWG and FRG groups. CAT activity was found to be decreased in the FRG and SEFRG groups compared to the SWG and SEG groups (Table 2). It was determined that GR activity was increased in FRG group compared to SWG group. There were no significant differences in GR activities in the SEG and SEFRG groups. G6PD activity decreased in FRG compared to SWG and increased in SEFRG compared to SEG groups. It was determined that there was a decrease in ROS value and LDH activity in SWG and SEG compared to FRG and SEFRG groups. Arginase activity decreased in SWG and SEG compared to FRG and SEFRG groups. Lipase activity decreased in the FRG and SEFRG groups compared to SWG and SEG groups (Table 2).

**TABLE 2:** Biochemical parameters of liver tissues of all groups.

Biochemical parameters	SWG	FRG	SEG	SEFRG	*p_ANOVA_*
**Glutathione (nmol GSH/mg protein)** ^*^	49.92 ± 6.23	45.81 ± 6.45	72.09 ± 5.69^b^	64.84 ± 5.93^b^	<0.0416
**Lipid peroxidation (nmol MDA/mg protein)** ^*^	11.55 + 1.73	11.56 ± 2.55	17.57 ± 3.08^g^	19.29 ± 1.10^a^	<0.0017
**Catalase (U/mg protein)** ^*^	1360.00 ± 94.6^d^	1220.00 ± 13.8	1121.00 ± 38.4e^h^, ^i^	725.90 ± 21.79^c^	<0.0001
**Superoxide dismutase (U/mg protein)** ^*^	37.12 ± 4.67	40.10 ± 6.43	47.99 ± 3.38^g^	43.84 ± 9.03	<0.1265
**Glutathione peroxidase (U/mg protein)** ^*^	64.91 ± 2.99^a^	42.05 ± 6.42	75.10 ± 12.48^f^	57.85 ± 11.22^d^	<0.0171
**Glutathione reductase (mU/g protein)** ^*^	14.55 ± 2.15	18.01 ± 2.06	13.94 ± 2.67	13.91 ± 0.87^d^	<0.1035
**Glutathione‐S‐transferase (U/g protein)** ^*^	48.30 ± 6.47	44.51 ± 4.96	44.80 ± 11.06	40.51 ± 7.54	<0.3544
**Glucose‐6‐phoshate dehydrogenase (U/g protein)** ^*^	50.35 ± 6.84^a^	35.30 ± 1.98	27.08 + 2.81^f^, ^h^	37.12 ± 5.65	<0.0448
**Reactive oxygen species (RFU/mg protein)** ^*^	1.86 ± 0.94^a^	6.39 ± 1.96	1.62 ± 0.36^e^	6.78 ± 2.18	<0.0094
**Lactate dehydrogenase (U/mg protein)** ^*^	6.32 ± 0.24	7.68 ± 0.60	5.13 ± 1.69	5.97 ± 0.76^d^	<0.2961
**Arginase (µmol urea/mg protein)** ^*^	0.75 ± 0.12^b^	1.81 ± 0.56	0.98 ± 0.02	1.15 ± 1.12	<0.0002
**Lipase (U/mg protein)** ^*^	3.88 ± 0.60^b^	2.66 ± 0.30	2.55 ± 0.21^i^	2.22 ± 0.49	<0.0001

Abbreviations: ANOVA, analysis of variance; FRG, Feeding restricted group; SEFRG, swimming exercised‐feed restricted group; SEG, swimming exercised group; SWG, static water group.

* Mean ± SD.

^a^ *p* < 0.01 vs. FRG.

^b^ *p* < 0.001 FRG.

^c^ *p* < 0.0001 vs. FRG.

^d^ *p* < 0.05 vs. FRG.

^e^ *p* < 0.01vs. SEFRG.

^f^ *p* < 0.05 vs. SEFRG.

^g^ *p* < 0.01 vs. SWG.

^h^ *p* < 0.0001 vs. SWG.

^i^ *p* < 0.0001vs. SEFRG.

^j^ *p* < 0.05 vs. SWG.

It was determined that there was a decrease in LPO value in SEG and SWG groups compared to SEFRG and FRG groups in kidney tissues. Glutathione levels and CAT, SOD, GPx, GR, GST and G6PD activities were decreased in FRG and SEFRG group compared to SWG and SEG groups. It was observed that the ROS value increased in the FRG group compared to SWG and decreased in the SEFRG group compared to the SEG groups. It was observed that LDH activity increased in the SWG group compared to FRG group and decreased in the SEG group compared to SEFRG groups. Arginase activity decreased in FRG and SEFRG group compared to SWG and SEG groups (Table 3).

**TABLE 3:** Biochemical parameters of kidney tissues of all groups.

Biochemical parameters	SWG	FRG	SEG	SEFRG	*p_ANOVA_*
**Glutathione (nmol GSH/mg protein)** ^*^	1.50 ± 0.22	1.28 ± 0.15	1.46 ± 0.20	1.25 ± 0.16	˂0.1346
**Lipid peroxidation (nmol MDA/mg protein)** ^*^	7.60 ± 0.31^d^	9.88 ± 0.40	7.10 ± 0.05^i^	8.71 ± 0.11^b^	˂0.0001
**Catalase (U/mg protein)** ^*^	6.39 ± 009^d^	2.32 ± 0.06	5.42 ± 0.04	4.35 ± 0.40^d^	˂0.0001
**Superoxide dismutase (U/mg protein)** ^*^	144.40 ± 35.1^e^	97.69 ± 4.22	72.33 ± 15.76^g^	52.66 ± 5.65^e^	˂0.0001
**Glutathione peroxidase (U/mg protein)** ^*^	3.25 ± 0.35^e^	2.77 ± 0.20	2.44 ± 0.05^c^	2.26 ± 0.17^e^	˂0.0001
**Glutathione reductase (mU/g protein)** ^*^	25.14 ± 2.74^a^	14.54 ± 2.27	26.26 ± 3.57	24.72 ± 3.14^a^	˂0.0003
**Glutathione‐S‐transferase (U/g protein)** ^*^	106.40 ± 19.09^d^	39.51 ± 5.00	51.18 ± 13.95f^c^, ^f^	27.41 ± 4.47	˂0.0001
**Glucose‐6‐phosphate dehydrogenase (U/g protein)** ^*^	3.00 ± 0.51^d^	1.50 ± 0.27	1.89 ± 0.40^h^, ^i^	0.58 ± 0.09^b^	˂0.0001
**Reactive oxygen species (RFU/mg protein)** ^*^	17.46 ± 0.50^b^	43.51 ± 8.93	16.17 ± 2.72	14.79 ± 6.20^d^	˂0.0001
**Lactate dehydrogenase (U/mg protein)** ^*^	2.06 ± 0.76	1.90 ± 0.25	0.94 ± 0.33^d^	1.25 ± 0.44^e^	˂0.0001
**Arginase (µmol urea/mg protein)** ^*^	24.93 ± 1.17	23.43 ± 4.34	19.91 ± 2.30^h^	17.01 ± 1.05^b^	<0.0001

Abbreviations: ANOVA, analysis of variance; FRG, Feeding restricted group; SEFRG, swimming exercised‐feed restricted group; SEG, swimming exercised group; SWG, static water group.

* Mean ± SD.

^a^ *p* < 0.01 vs. FRG.

^b^ *p* < 0.001 vs. FRG.

^c^ *p* < 0.001 vs. SWG.

^d^ *p* < 0.0001vs. FRG.

^e^ *p* < 0.05 vs. FRG.

^f^ *p* < 0.05 vs. SEFRG.

^g^ *p* < 0.01 vs. SWG.

^h^ *p* < 0.0001 vs. SWG.

^i^ *p* < 0.0001 vs. SEFRG.

## 4 DISCUSSION

Glutathione functions as an endogenous protective system, safeguarding cells against damage induced by ROS, and is notably abundant in various tissues, particularly the liver. It reacts with ROS and peroxides, providing a defence against oxidative stress (Frei, 1994; Yalçın, 1998). With a tripeptide structure, glutathione (γ‐glutamate‐cysteine‐glycine) stands out as one of the most potent non‐enzymatic molecules for combating oxidative damage. Its robust antioxidant properties play a crucial role in mitigating the detrimental effects of ROS, making it a pivotal molecule in averting potential cell damage. Consequently, in studies on oxidative damage, it becomes imperative to assess GSH levels, as elevated GSH levels indicate the potential presence of oxidative damage (Srikanth et al., 2013). Malnutrition and stress lead to a decline in the body's GSH levels. In our study, a notable increase in GSH levels was observed in the stomach, liver and kidney tissues of fish subjected to abundant feeding in both static and flowing waters, as opposed to those with restricted feeding. Evidently, the antioxidant system responds to nutritional and exercise influences, consequently enhancing fish growth and development. Notably, the study unveiled augmented GSH levels in stomach, liver and kidney tissues among fish subjected to ample feeding, reinforcing the notion of nutrition and exercise‐induced antioxidant system elevation, thereby fostering fish growth and development.

With the rise in the concentration of highly reactive ROS within tissues, lipid oxidation takes place. Elevated levels of LPO lead to a reduction in membrane fluidity and, subsequently, a decline in the activity of membrane‐bound enzymes. Consequently, heightened oxidative stress induces an increase in LPO levels, contributing to the deterioration of the cell membrane (Powers et al., 2020). Engaging in exercise may intensify the generation of free radicals through accelerated metabolic processes (Palmer et al., 2003). In such cases, the surplus free radicals may evade elimination by antioxidant defence mechanisms, potentially triggering LPO chain reactions (Duthie et al., 1990).

Research indicates that oxidative stress and LPO elevate proportionately with vigorous and rapid exercises (Goldfarb, 1993). In a healthy organism, a balance exists between the oxidant and antioxidant systems. Swimming performance is governed by a plethora of biological and physiological determinants, further impacting various enzyme activities and non‐enzymatic factors influencing swimming performance. Our investigation exhibited no substantial increase in LPO values within stomach tissues, a decrement within kidney tissues, and no variation within liver tissues of fish with normal feeding as compared to those with restricted feeding in stationary water. This phenomenon suggests that enhanced nutrition corresponds to a reduction in LPO through heightened antioxidant activity, signifying a beneficial outcome of exercise in mitigating oxidative stress over extended periods.

Appropriate physical exercises can significantly contribute to maintaining a robust lifestyle (Vincent et al., 2002). Fish, akin to other vertebrates, possess an antioxidant defence system pivotal in counteracting the deleterious effects of hydrogen peroxide and ROS, implicated in oxidative harm to cellular components such as lipids, proteins and DNA, which may culminate in cellular demise (Yonar & Sakin, 2011). This defence mechanism encompasses both enzymatic and non‐enzymatic antioxidants, including SOD, catalase, GPx, GST, G6PD and GR (Blahová et al., 2013; Droge, 2002). These systems collaborate to scavenge free radicals and preclude the generation of ROS, thereby mitigating oxidative damage (Aydın et al., 2001; Li et al., 2009). The functioning of antioxidant enzymes, such as CAT, SOD, GPx, GR and GST, is intricately linked to the antioxidant status of tissues. These enzymes provide valuable insights into the level of oxidative stress and furnish information about the extent of oxidative damage. As oxidative stress occurs, the generation of superoxide radicals in the organism escalates. SOD transforms superoxide radicals into H_2_O_2_, and CAT and/or GPx further convert H_2_O_2_ into H_2_O. The elevation of GPx activity leads to the formation of oxide glutathione (GSSG), which, with the assistance of the GR enzyme, synthesizes GSH. This orchestrated action by the antioxidant enzyme system serves to minimize the adverse effects of ROS in the organism (Irato & Santovito, 2021). GR, an antioxidant enzyme pivotal in reducing GSSG to two molecules of GSH, relies on nicotinamide adenine dinucleotide phosphate (NADPH) for its activity. Consequently, the activity of GR is closely associated with that of G6PD activity (Ho et al., 2007). Any increase in the levels of ROS may instigate changes in the activities of the enzymes comprising this system. Therefore, the determination of antioxidant enzyme activities, interconnected with the assessment of oxidative stress, holds a significant role in comprehending tissue damage. A disparity in antioxidant and oxidant levels may lead to reduced or imbalanced antioxidant enzyme activities due to an accumulation of excessive ROS, consequently eliciting oxidative stress within the organism (Valavanidis et al., 2006). The antioxidant defence system in fish is intricately entwined with environmental conditions, dietary habits and nutritional constituents. The evaluation of antioxidant enzymes and substances stands as a requisite in assessing fish well‐being (Sagstad et al., 2007). Consequently, the interaction between the antioxidant system, ROS formation, swimming performance and nutritional deficits in fish becomes evident.

Swimming activity and feed restriction emerge as determinants influencing enzyme activities and antioxidant systems within fish tissues. Remarkably, exercise and improved nutrition are seen to amplify antioxidant enzyme activities while curbing LPO. Noteworthy, the impact of swimming performance and nutritional inadequacies on the antioxidant system and ROS formation within fish is striking. Moreover, an escalation in the efficiency of LDH enzyme, crucial in the final phase of glycolysis across living organisms, denotes tissue impairment.

In situations characterized by anaerobic or hypoxic conditions, oxidative phosphorylation experiences a slowdown or complete disruption. Consequently, an alternate energy metabolism pathway becomes activated, leading to an upregulation in the activity of LDH in the liver. Scrutinizing this adaptive response yields valuable insights into tissue energy metabolism. Although LDH does not function as a direct antioxidant enzyme, its activity and release may be influenced by oxidative stress, serving as an indicator of cellular damage linked to oxidative imbalance. In instances of oxidative damage, cellular oxygen levels decrease, prompting the initiation of alternative pathways such as gluconeogenesis to sustain cellular energy needs. l‐lactate, particularly generated through LDH activity, can play a role in glucose production, contributing to energy regeneration via gluconeogenesis. Consequently, an indirect relationship exists between oxidative stress and LDH activity in the context of cellular energy adaptation and damage assessment (Sacheck & Blumberg, 2001). Nonetheless, although exercise remains essential for well‐being, moderate physical activity may lead to muscle loss, heightened oxidative stress and inflammation (Malaguti et al., 2013). The detection of cellular damage can be facilitated through LDH activity determination, signifying a pivotal diagnostic measure. The tandem of exercise and a wholesome diet assumes significance, with an increased LDH level being attributed to exercise. In this context, an elevation in LDH activity was identified in gastric tissues due to overnutrition and exercise.

Although the carbon skeleton is used as an energy source, especially as a result of the digestion of proteins, the NH_3_ formed as a result of hydrolysis is quite reactive and must be converted into urea, a more non‐toxic form. For this purpose, NH_3_ transported to the liver is included in the urea cycle and converted into urea. One of the key enzymes in the urea cycle is arginase. Arginase, a binuclear manganese metalloenzyme in urea, catalyses the hydrolysis of l‐arginine into urea and l‐ornithine, and the resulting urea is excreted from the organism (Li et al., 2022). The scope of arginase enzyme activity encompasses both ureotelic and non‐ureotelic organisms (Barim & Erisir, 2009). In non‐ureotelic organisms, arginase plays a role not only in the urea cycle but also in protein metabolism, creatinine synthesis, nitric oxide generation and polyamine biosynthesis (Jenkinson et al., 1996). The arginase enzyme's activity profile is subject to nutritional influences. Research indicates that low‐protein diets lead to reduced urea cycle enzyme activities, a trend reversed upon increased protein intake (Shombough, 1978). Moreover, carbohydrate‐rich diets were observed to diminish urea cycle enzyme activities, contrasting with the elevation seen during carbohydrate‐restricted diets (Schimke, 1962). The absence of this enzyme is associated with growth suppression (Grillo & Colombatto, 2004). Arginine degradation to urea and ornithine via arginase in fish is modulated by dietary arginine levels. The quantity of urea, ornithine and hepatic arginase activity directly correlates with dietary arginine levels. An increase in arginase activity was observed in the liver tissue of fish with decreased nutritional value, particularly in both stationary and flowing water conditions, and in the kidney tissue of fish exposed to overnutrition.

The activity of enzymes in fish's digestive system varies based on factors, such as organ location, season, diurnal rhythm, satiety status, feeding frequency, diet and age (Furné et al., 2008; Yüngül & Özdemir, 2017). Fish growth is closely linked to feed conversion efficiency and digestive enzyme augmentation. Digestive enzymes play a vital role in hydrolysing ingested food, thus facilitating nutrient absorption (Trestrail et al., 2021). There are different enzymes for the digestion of various nutrients, and these macromolecules are digested in different tissues. Specifically, when proteins are not consumed in sufficient levels, organisms begin to hydrolyse their proteins to sustain life. This situation impacts vital functions and may also lead to an increase in enzyme activities (such as protease and pepsin) involved in protein digestion. Similarly, the breakdown of fat molecules, which serve as energy sources, and their relationship with energy metabolism may affect enzyme activities involved in fat digestion (Rothman et al., 2002). Therefore, it can be inferred that there is a relationship between food deprivation, exercise and the activities of digestive enzymes.

The analysis of enzymatic activities responsible for protein and fat hydrolysis in this study elucidates the effects of swimming activity and feed restriction on the development of fish. Existing research underscores that exercise can confer both positive and detrimental impacts on organisms. An adverse consequence involves the generation of free radicals and LPO due to exercise (Gonenc, 1995). The exercise intensity directly influences free radical generation, with greater intensity exercises yielding higher levels of free radicals (Palmer et al., 2003). Intense exercises are linked to heightened ROS and free radical generation, triggering oxidative impairment in muscles, liver, blood and other tissues (König et al., 2001; Urso & Clarkson, 2003). Regular and extended exercises are posited to fortify the ‘antioxidant defence system’, concurrently diminishing LPO products (Ji, 1999; Gul et al., 2001). Effective exercise, therefore, contributes to sustaining a wholesome lifestyle (Ralph et al., 2012; Vincent et al., 2002).

## 5 CONCLUSIONS

In conclusion, this study highlights the complex interactions between swimming activity, feed restriction and antioxidant enzyme activities in rainbow trout. The results suggest that swimming activity and proper nutrition contribute to enhancing the antioxidant defence system and promoting fish growth and development. The findings emphasize the importance of balanced nutrition and exercise in aquaculture practices to maintain fish health and improve performance. However, further research is needed to understand the specific mechanisms underlying these effects and to optimize exercise regimes and dietary strategies for fish rearing in RAS.

## Summary

This study delves into the effects of swimming activity and feed restriction on the digestion and antioxidant enzyme activities in juvenile rainbow trout with an average body weight of 26.54 ± 0.36 g. Four distinct groups were examined: Static Water Group (SWG), Feeding Restricted Group (FRG), Swimming Exercised Group (SEG) and Swimming Exercised‐Feed Restricted Group (SEFRG). The research focused on assessing glutathione levels, lipid peroxidation and the activities of various enzymes, such as catalase, superoxide dismutase, glutathione peroxidase, glutathione reductase, glucose‐6‐phosphate dehydrogenase and lactate dehydrogenase. Reactive oxygen species presence and the activities of pepsin, protease, lipase and arginase were also measured in the stomach, liver and kidney tissues. The findings suggest that proper nutrition and exercise positively impact the fish's antioxidant system, reducing free radical formation and contributing to overall fish development. This study holds significance for understanding the interplay between exercised fish, restricted feeding, enzyme activity and animal welfare in recirculating aquaculture systems.

## AUTHOR CONTRIBUTIONS

*Formal analysis; investigation; data curation; writing – original draft; writing – review and editing; visualization*: Gökhan Tunçelli. *Investigation; data curation; writing – review and editing*: Onur Ertik. *Investigation; data curation; writing – review and editing*: Bertan Boran Bayrak. *Conceptualization; methodology; validation; resources; writing – review and editing; supervision; project administration*: Devrim Memiş. *Data curation; writing – review and editing; supervision*: Refiye Yanardag.

## CONFLICT OF INTEREST STATEMENT

The authors declare that they have no conflicts of interest.

## ETHICS STATEMENT

The authors confirm that the ethical policies of the journal, as noted on the journal's author guidelines page, have been adhered to and the appropriate ethical review committee approval has been received. The authors confirm that they have followed EU standards for the protection of animals used for scientific purposes. The present experiments were carried out according to the legislation on the use of aquatic animals for experimental and other scientific purposes in Turkey with approval of Istanbul University, Animal Experiments Local Ethics Committee (Number: 2019/10).

## Data Availability

The data that support the findings of this study are available on request from the corresponding author. The data are not publicly available due to privacy or ethical restrictions.
